# PPAR-**α** Agonist Fenofibrate Upregulates Tetrahydrobiopterin Level through Increasing the Expression of Guanosine 5′-Triphosphate Cyclohydrolase-I in Human Umbilical Vein Endothelial Cells

**DOI:** 10.1155/2011/523520

**Published:** 2011-11-16

**Authors:** Jinbo Liu, Changlin Lu, Fuwang Li, Haining Wang, Liyun He, Yanting Hao, Alex F. Chen, Huijie An, Xian Wang, Tianpei Hong, Guang Wang

**Affiliations:** ^1^Department of Endocrinology, Peking University Third Hospital, Beijing 100191, China; ^2^Department of Cardiology, Beijing Tongren Hospital, Beijing 100191, China; ^3^Department of Physiology and Pathophysiology, School of Basic Medical Sciences and Key Laboratory of Molecular Cardiovascular Science of Ministry of Education, Peking University Health Science Center, Beijing 100191, China; ^4^Department of Cardiovascular Medicine, Peking University Third Hospital, Beijing 100191, China; ^5^Department of Surgery, University of Pittsburgh School of Medicine and Vascular Surgery Research, Pittsburgh, PA 15240, USA

## Abstract

Tetrahydrobiopterin (BH4) is an essential cofactor for endothelial nitric oxide (NO) synthase. Guanosine 5′-triphosphate cyclohydrolase-I (GTPCH-I) is a key limiting enzyme for BH4 synthesis. In the present in vitro study, we investigated whether peroxisome proliferator-activated receptor **α** (PPAR-**α**) agonist fenofibrate could recouple eNOS by reversing low-expression of intracellular BH4 in endothelial cells and discussed the potential mechanisms. After human umbilical vein endothelial cells (HUVECs) were treated with lipopolysaccharide (LPS) for 24 hours, the levels of cellular eNOS, BH4 and cell supernatant NO were significantly reduced compared to control group. And the fluorescence intensity of intracellular ROS was significantly increased. But pretreated with fenofibrate (10 umol/L) for 2 hours before cells were induced by LPS, the levels of eNOS, NO, and BH4 were significantly raised compared to LPS treatment alone. ROS production was markedly reduced in fenofibrate group than LPS group. In addition, our results showed that the level of intracellular GTPCH-I detected by western blot was increased in a concentration-dependent manner after being treated with fenofibrate. These results suggested that fenofibrate might help protect endothelial function and against atherosclerosis by increasing level of BH4 and decreasing production of ROS through upregulating the level of intracellular GTPCH-I.

## 1. Introduction

Nitric oxide (NO) is a gas with a half-life of several seconds. It has a pivotal role in vascular homeostasis, such as regulating vascular tone and blood pressure inhibiting platelet aggregation and proliferation of vascular smooth muscle cells and the expression of inflammatory cytokines. Many studies have established that loss of NO bioavailability is a key feature of endothelial dysfunction preceding the appearance of atherosclerosis [1, 2].

Endogenous NO is derived from L-arginine in the catalysis of endothelial NO synthase (NOS), which has binding sites for tetrahydrobiopterin (BH4), L-arginine, heme, flavin adenine dinucleotide (FAD), and flavin mononucleotide (FMN) [3]. BH4 is an essential cofactor for endothelial NOS (eNOS). It not only stabilizes the structure of eNOS but also plays a role in the electron transfer during the production of NO [4, 5]. When BH4 levels are inadequate, eNOS is no longer coupled to L-arginine oxidation, which results in reactive oxygen species (ROS) rather than NO production, thereby inducing vascular endothelial dysfunction [6, 7]. Several studies suggest a possible role for BH4 in regulating NO-mediated endothelial function. Oral administration of BH4 can improve endothelial function in hypercholesterolemia patients, diabetic patients, and patients with or without coronary artery disease (CAD) [8–11]. Ozaki et al. showed that chronic overexpression of eNOS does not inhibit, but accelerates, atherosclerosis under hypercholesterolemia and that eNOS dysfunction appears to play important roles in the progression of atherosclerosis in apoE-deficient/eNOS-overexpressing mice (apoE-KO/eNOS-Tg mice). But supplementation with BH4 reduced the atherosclerotic lesion size in apoE-KO/eNOS-Tg mice to the level comparable to apoE-KO mice [12]. Another study showed that oral administration of BH4 slowed the progression of atherosclerosis in apoE-KO mice [13]. Guanosine 5′-triphosphate cyclohydrolase-I (GTPCH-I), encoded by the GCH-I gene, is the rate-limiting enzyme in BH4 synthesis. In diabetic GCH-Tg mice, superoxide production from endothelium was markedly reduced compared with that of diabetic mice induced by streptozotocin, and NO-mediated vasodilatation was preserved [14]. Zheng et al. [15] found that gene transfer of GTPCH-I could reverse the BH4 deficiency and endothelial dysfunction by reducing O_2_^−^ in low renin mineralocorticoid hypertension. In addition, exposure of bovine aortic endothelial cells to GTPCH-I inhibitors or siRNA markedly reduced BH4 and NO levels but increased superoxide anion levels [16]. 

Hypolipidemic fibrates are pharmacological compounds which activate peroxisome proliferator-activated receptor *α* (PPAR-*α*), a number of the nuclear hormone receptor superfamily. Many studies showed that PPAR-*α* agonists have a lot of benefits on endothelial function independent of their hypolipidemic effects. Researchers found that PPAR-*α* activators play a role in cardiovascular protection through an increase in eNOS expression level and phosphorylation of eNOS ser-1179 and NO production in bovine aortic endothelial cells [17, 18]. Ryan et al. reported that PPAR-*α* agonist fenofibrate improves endothelial function and reduces arterial stiffness in obese glucose-tolerant men [19]. Recent evidence indicates that recoupling eNOS may be a potential approach augmenting the effects of PPAR-*α* on eNOS. However, the precise effects of PPAR*α* agonist on recoupling eNOS and its potential mechanism remain uncertain.

Our previous study demonstrated that homocysteine impairs coronary artery endothelial function by decreasing the level of BH4 in patients with hyperhomocysteinemia [20]. Our previous study also showed that plasma level of BH4 was significantly increased by PPAR*α* agonist fenofibrate in patients with hypertriglyceridemia. Moreover, coronary flow velocity reserve (CFVR) was significantly improved with fenofibrate treatment [21].

Despite PPAR-*α* activation may have favorable endothelium-protecting properties, the precious mechanism on eNOS coupling status remains uncertain. In the present study, we investigated whether PPAR-*α* agonist fenofibrate could improve the expression of intracellular BH4 through upregulating GTPCH-I, thus contributing to the recoupling of eNOS.

## 2. Materials and Methods

### 2.1. Cell Culture

Endothelial cells were isolated from segments of human umbilical cord vein by collagenase digestion. They were cultured in medium 199 supplemented with 10% fetal calf serum as previously described [22]. The medium was renewed every 2 days until confluence (3-4 days); cells were then detached by incubation in PBS containing 0.05% trypsin and 0.03% EDTA for 1 min at room temperature, washed by centrifugation and reseeded onto 35, 60, or 100 mm plastic culture dishes for ROS, detection, eNOS, BH4, and GTPCH-I measurement. At early confluence, cells were treated with LPS in the presence of fenofibrate or not as indicated in the figure legends. Only endothelial cells passaged less than six times were used for experiments. 

### 2.2. Measurement of Intracellular BH4

For the measurement of total biopterin, high-performance liquid chromatography (HPLC) was used, as previously described with some modification [23]. Cell lysates were suspended in distilled water containing 1 mM Dithiothreitol, 50 mM Tris-HCl (pH 7.4), and 1 mM EDTA, centrifuged at 12000 g at 4°C for 15 min, and then subjected to oxidation in acid and base. The supernatant (90 ul) was transferred to an amber tube, and 10 uL of 1 : 1 mixture of 1.5 M HClO4 and 2M H3PO4 was added, followed by centrifugation at 13000 g for 10 min at 4°C. The supernatant (90 ul) was transferred to a new amber tuber, and 10 uL of iodine solution (1% iodine and 2% KI in 1 M HCl solution) was added to the process of acid oxidation in order to determine total biopterin (BH4, dihydropterin (BH2), and oxidized biopterin(B)). After mixing and standing for 60 min in the dark at room temperature, excess iodine was reduced by the addition of 5 uL fresh ascorbic acid (20 mg/mL in water). To determine BH2 + B by alkaline oxidation, 10 uL of 1 M NaOH was added to 80 uL extract, and then 10 uL of alkaline iodine solution (1% iodine and 2% KI in 1 M NaOH solution) was added. After mixing and standing for 60 min in the dark at room temperature, 20 uL of 1 M H_3_PO_4_ was added to acidify alkaline oxidation, and then 5 uL fresh ascorbic acid (20 mg/mL in water) was added to reduce excess iodine. Samples oxidized under acidic or alkaline conditions were centrifuged at 13000 g for 10 min at 4°C. The supernatant 90 uL was injected into the column by use of an HPLC system with an autosampler and a fluorescence detector (Agilent 1100). A Hypersil C18 column (4.6 mm × 250 mm, 5 um) was used for separation of biopterin with a mobile phase of ration of methanol to water (5 : 95, v/v) running at a flow rate of 1.0 mL/min. The retention time of biopterin was approximately 7.5 min, and the excitation and emission wave lengths were 350 and 440 nm, respectively. Compounds were quantitated by their peak height in comparison with external standards. And BH4 concentrations, expressed as pmol/mg protein, were calculated by subtracting BH2 + B from total biopterin. 

### 2.3. Measurement of Intracellular eNOS

Level of eNOS was measured by use of ELISA kits according to the manufacturer's protocols (BioPCR, China).

### 2.4. Measurement of Cell Supernatant NO

NO level was measured by use of an ELISA kit according to the manufacturer's protocols (Jiamay Biotech, China).

### 2.5. Measurement of Intracelluar ROS Generation

Determination of intracellular oxidant production in endothelial cells was based on the oxidation of an ROS probe dye 2′,7′-dichlorofluorescin diacetate (DCF-DA, 20 umol/L) by intracellular ROS, and resulting in the formation of the fluorescent compound 2′,7′-dichlorofluorescin (DCF). And DCF florescence was monitored with a confocal laser scanning microscope (Leica) [24].

### 2.6. Western Blot Analysis

HUVECs were lysated with cell-lysis buffer (150 mM NaCl, 100 mM Tris-HCl pH 7.4, 1 mM Na_2_ EDTA, 1% Triton-X, 10 ug/mL aprotinin, 10 ug/mL pepstatin A, 10 ug/mL leupeptin, 0.05 M NaF, 0.01 M Na_4_O_7_P_2_, 1 M Na_3_VO_4_) and 1 mM PMSF. The protein content was assayed by BCA protein assay reagent. 40 ug protein were loaded to SDS-PAGE and then transferred to PVDF membranes. After incubation for 1 hour in blocking buffer (5% skim milk powder in TBS-T), the membranes were incubated with primary antibody (Santa Cruz, USA) with a 1 : 1000 dilution, followed by incubating with fluorescently labeled secondary antibody (Invitrogen, USA). Protein bands were visualized by Odyssey infrared imaging system (LI-COR Biosciences, USA), and the intensity of each bands was measured by the accompanied software. We used control as 100%.

### 2.7. Statistical Analysis

Dates were reported as means ± SD. The differences between groups were analyzed by one-way ANOVA, and *P* < 0.05 was considered statistically significant.

## 3. Results

### 3.1. Effects of Fenofibrate on Intracellular eNOS and Cell Supernatant NO

**Figure 1:**
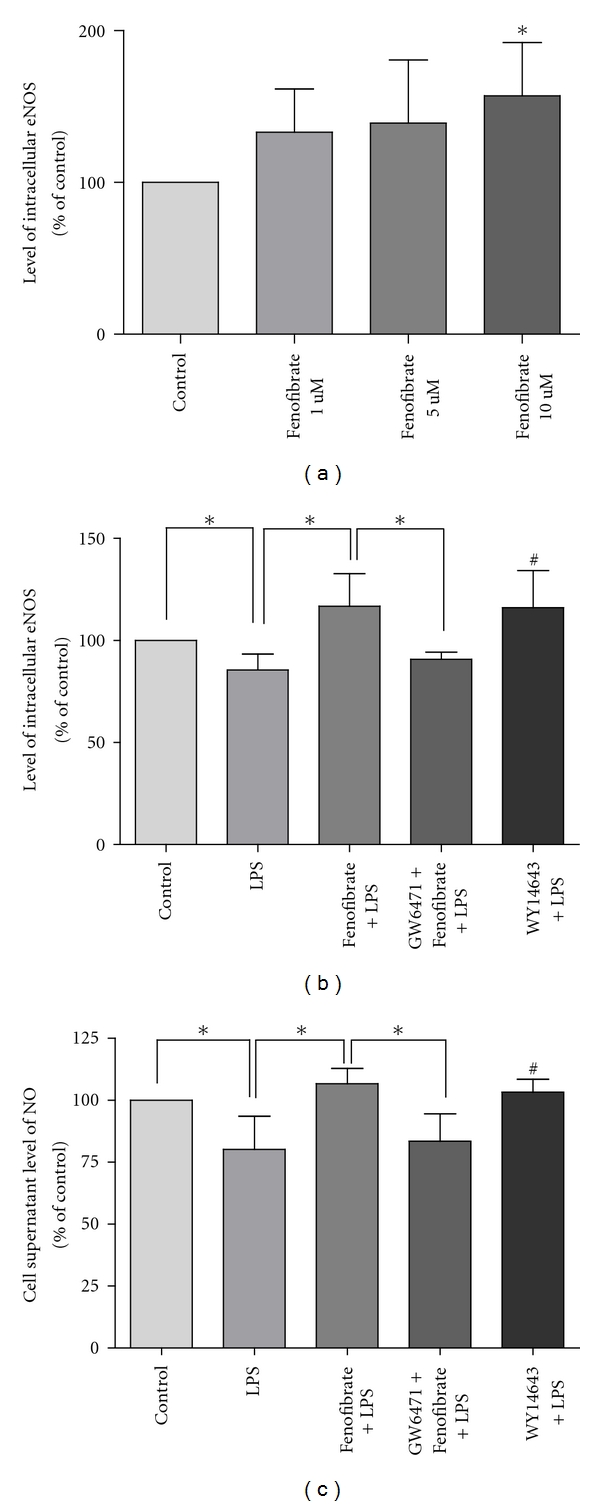
Effect of fenofibrate and related compounds on levels of eNOS (a, b) and NO (c) in control group, LPS group, fenofibrate pretreated group, PPAR*α* antagonist, and agonist group. eNOS and NO were measured by ELISA kits according to the manufacturer's protocols. Values are expressed as means ± SD. (a) *P < 0.05 versus control group. (b, c) *P < 0.05; ^#^ *P* < 0.05 versus LPS group.

As shown in Figure 1, fenofibrate treatment for 24 hours increased the expression of eNOS protein in a concentration-dependent manner. However, after HUVECs were treated with LPS for 24 hours, the levels of cellular eNOS and cell supernatant NO were significantly reduced by 14.4%, 19.9% (both *P* < 0.05) compared to control groups, respectively. But pretreated with fenofibrate (10 umol/L) for 2 hours before cells were induced by LPS, the levels of eNOS and NO were significantly raised compared to LPS treatment (both *P* < 0.05). We also found that PPAR*α* blocker GW6471 (10 umol/L) abolished the effect of fenofibrate and PPAR*α* agonist WY14643 (50 umol/L) could produce a similar effect of fenofibrate on intracellular eNOS and cell supernatant NO. And there was no significant difference between group of WY14643 + LPS and group of fenofibrate + LPS in the levels of eNOS and NO (*P* = 0.93, *P* = 0.12, resp.).

### 3.2. Effect of Fenofibrate on Intracellular Level of ROS

**Figure 2:**
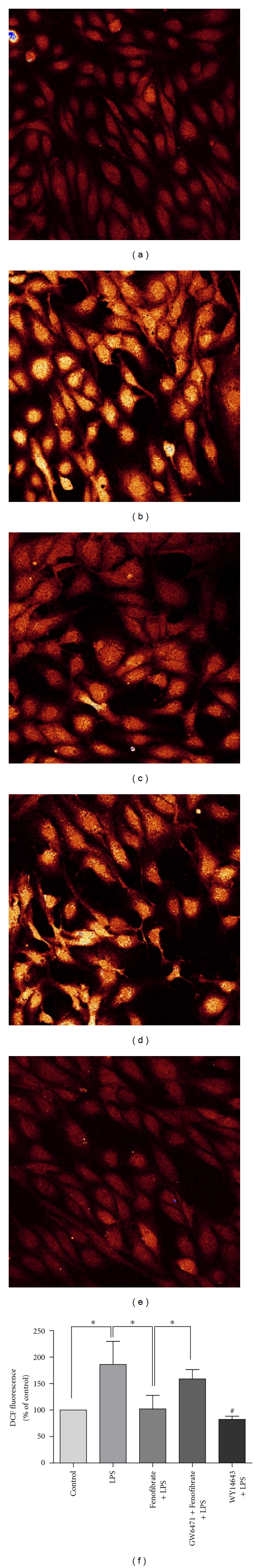
Effect of fenofibrate and related compounds on ROS formation in cultured endothelial cells. DCFH-DA (20 umol/L) was added to monitor intracellular ROS production with a confocal laser scanning microscope (Leica). (a) Control group; (b) LPS group; (c) fenofibrate + LPS; (d) GW6471 + fenofibrate + LPS; (e) WY14643 + LPS; (f) statistical graph. Values are expressed as means ± SD; *n* = 6. *P < 0.05; ^#^ *P* < 0.05 versus LPS group.

As shown in Figure 2, the fluorescence intensity of intracellular ROS was stronger in LPS group. However, if cells were pretreated with fenofibrate for 2 hours before being incubated with LPS for 24 hours, ROS fluorescence intensity was significantly reduced in fenofibrate group than LPS group. In addition, the fluorescence intensity was stronger than fenofibrate group after being treated by PPAR*α* antagonist GW6471 (10 umol/L). We also found that PPAR*α* agonist WY14643 (50 umol/L) could reduce the level of intracellular ROS similarly to the effect of fenofibrate. And there was no significant difference between group of WY14643 + LPS and group of fenofibrate + LPS in the level of ROS (*P* = 0.25).

### 3.3. Effect of Fenofibrate on Intracellular Level of BH4

**Figure 3:**
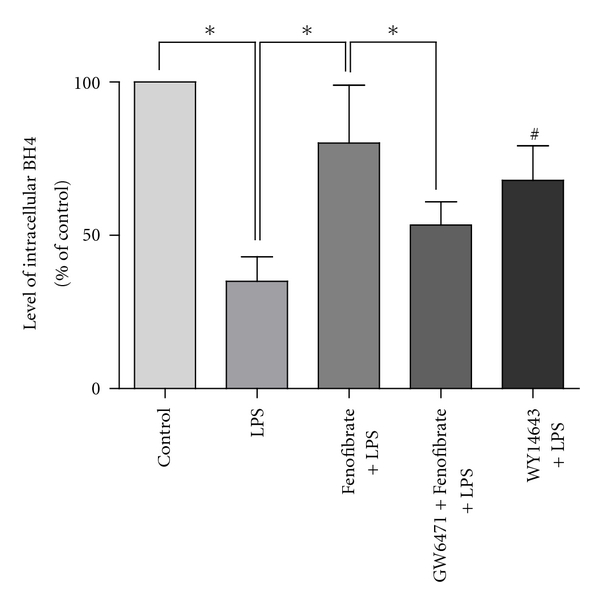
Effect of fenofibrate and related compounds on intracellular level of BH4 in each group, measured by HPLC. Values are expressed as means ± SD. BH4: tetrahydrobiopterin. *n* = 5. *P < 0.05; ^#^ *P* < 0.05 versus LPS group.

BH4 is an essential cofactor for eNOS. When BH4 levels are inadequate, eNOS is no longer coupled to L-arginine oxidation, which results in ROS rather than NO production. From above statements we knew that LPS stimulation could cause more production of ROS in HUVECs, so we next investigated whether the action of LPS was from the pathway of BH4. As shown in Figure 3, intracellular BH4 level was significantly lower in LPS group than control group (*P* < 0.05). However, pretreated with fenofibrate (10 umol/L) for 2 hours, intracellular BH4 level was significantly higher than LPS group (*P* < 0.05). PPAR*α* blocker GW6471 (10 umol/L) abolished the effect of fenofibrate on intracellular BH4. And PPAR*α* agonist WY14643 (50 umol/L) could upregulate the level of intracellular BH4 similarly to fenofibrate without significant difference (*P* = 0.25).

### 3.4. Effect of Fenofibrate on Intracellular Level of GTPCH-I

**Figure 4:**
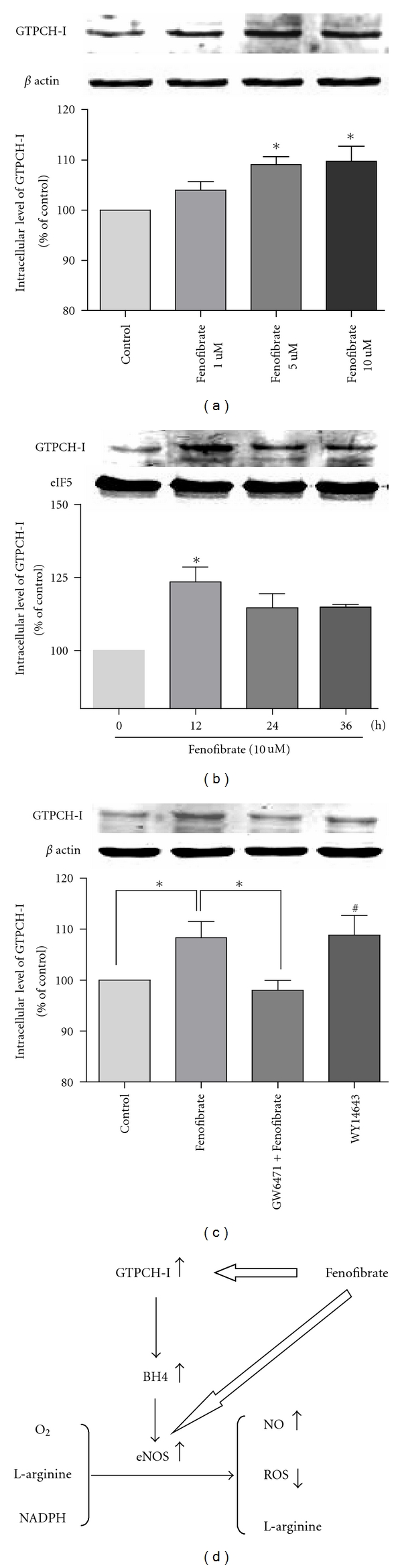
Fenofibrate increases GTPCH-I protein levels in HUVECs, as demonstrated by Western blot analyses. (a) Cells were treated for 24 h with increasing concentrations of fenofibrate. (b) The level of GTPCH-I after fenofibrate treatment reached maximum at 12 hours and maintained a high level for a long time. (c) GW6471 antagonizes fenofibrate-induced increase in GTPCH-I protein level, WY14643 increases GTPCH-I protein level similarly to the effect of fenofibrate. Dates are means ± SD in three experiments. (a, b): *P < 0.05 versus control group; (c) *P < 0.05, ^#^ *P* < 0.05 versus control group. (d) Proposed scheme: Fenofibrate not only promotes the expression of eNOS, but also up-regulates the level of GTPCH-I, contributing to the increase of BH4, which could promote the activity of eNOS, thus resulting the increase of NO and decrease of ROS.

GTPCH-I is the rate-limiting enzyme in BH4 synthesis. We next investigated whether fenofibrate promoted the expression of BH4 through increasing the level of GTPCH-I in HUVECs. As shown in Figure 4, fenofibrate treatment for 24 hours increased GTPCH-I expression in a concentration-dependent manner. The level of GTPCH-I after fenofibrate treatment reached maximum at 12 hours and maintained at a high level for a long time. We also found that PPAR*α* blocker GW6471 (10 umol/L) abolished the effect of fenofibrate and PPAR*α* agonist WY14643 (50 umol/L) could produce a similar effect of fenofibrate on intracellular GTPCH-I. And there was no significant difference between WY14643 group and fenofibrate group in the expression of GTPCH-I (*P* = 0.81).

## 4. Discussion

BH4 is an essential cofactor for eNOS which catalyzes L-arginine to L-citrulline with the production of NO, one of the most important vasodilators. Our study demonstrated that endothelial cellular eNOS and NO were significantly reduced when incubated with LPS, accompanied by more ROS production and lower level of BH4. However, when pretreated with fenofibrate before exposing to LPS, cellular levels of eNOS, NO, and BH4 were all significantly higher than LPS group. There was lower level of ROS in the fenofibrate group than LPS group. Fenofibrate treatment for 24 hours increased GTPCH-I expression in a concentration-dependent manner. Finally, PPAR*α* antagonist GW6471 could abolish the effects of fenofibrate on these indicators, and PPAR*α* agonist WY14643 could produce the similar effect of fenofibrate. Thus, fenofibrate may help protect endothelial function by promoting level of BH4 and decreasing production of ROS through increasing the level of intracellular GTPCH-I.

Endothelial-derived NO is essential in the maintenance of vascular homeostasis. It has many biological effects, such as suppressing platelet aggregation, leukocyte migration and celluar adhesion to the endothelium, and inhibiting inflammatory response and vascular smooth muscle cell proliferation. Hence, NO is an anti-atherosclerotic molecule. So loss of NO bioavailability is a key feature of endothelial feature of endothelial dysfunction preceding the appearance of atherosclerosis [1, 25]. Hypolipidemic fibrates are pharmacological compounds which activate PPAR-*α*, a number of the nuclear hormone receptor superfamily. Many studies showed that PPAR-*α* agonists have a lot of benefits on vascular endothelial function independent of their hypolipidemic effects. Researchers found that PPAR-*α* activators play a role in cardiovascular protection through an increase in eNOS expression level and phosphorylation of eNOS ser-1179 and NO production in Bovine aortic endothelial cells [17, 18]. In our present study, we showed that fenofibrate improved the levels of eNOS and NO in endothelial cells. Tetrahydrobiopterin (BH4) is an essential cofactor that maintains the normal function of eNOS. When BH4 level is inadequate, it could transform eNOS into an oxidant production of ROS. BH4 may improve NO-mediated endothelial function, thereby potentially reducing the development of atherosclerosis. Several studies showed that oral administration of BH4 can improve endothelial function in hypercholesterolemia patients, diabetic patients, and patients with or without coronary artery disease (CAD) [8–11]. Then, correction of endothelial dysfunction may explain in part the beneficial effect of PPAR-*α* agonist treatment. Our previous study demonstrated that PPAR-*α* agonist fenofibrate increased the plasma levels of BH4 and NO in patients with hypertriglyceridemia [21]. Moreover, homocysteine impairs coronary artery endothelial function by inhibiting tetrahydrobiopterin in patients with hyperhomocysteinemia [20]. We hypothesized that fenofibrate could promote the activity of eNOS through restoring the level of BH4 contributing to lower level of ROS. As mentioned earlier, when treated with fenofibrate, the intracellular level of BH4 was significantly improved compared to LPS treatment alone, accompanied by lower ROS generation. These results suggest that fenofibrate might have potentially beneficial effects in endothelial function by promoting the level of BH4. 

Next, GTPCH-I is the rate-limiting enzyme in BH4 synthesis. In many gene transfer animal models of GTPCH-I, such as diabetic GCH-Tg mice and DOCA-salt hypertension rat, overexpression of GTPCH-I could reverse the BH4 deficiency and endothelial dysfunction by reducing superoxide anion production and NO-mediated vasodilatation was preserved [14, 15]. Wang et al. [16] found that exposure of bovine aortic endothelial cells to GTPCH-I inhibitors or siRNA markedly reduced BH4 and NO levels but increased superoxide levels. Some drugs such as statin and metformin can improve endothelial function through increasing level of BH4 by different mechanisms, such as increasing the level of GTP cyclohydrolase I (GTPCH I), suppressing 26 s proteasome-mediated GTPCH I degradation to increase the level of BH4, and so on [26–28]. In our system, we discovered that fenofibrate treatment increased GTPCH-I expression in a concentration-dependent manner. Then, fenofibrate might play its endothelial protecting and antiatherosclerosis effect by up-regulating BH4 and GTPCH-I levels in HUVECs, which could provide a new perspective for the therapy of endothelial dysfunction (Figure 4(d)). However, the precise mechanism of this process needs to be further investigated.

In conclusion, fenofibrate contributes beneficial effect to prevent endothelial dysfunction from upregulating level of BH4 and decreasing production of ROS through the mechanism of increasing the level of intracellular GTPCH-I. Fenofibrate may help protect against atherosclerosis potential by promoting the re-coupling of eNOS with normalizing endothelial disorders.
